# Stratification of β^S^β^+^ Compound Heterozygotes Based on L-Glutamine Administration and RDW: Focusing on Disease Severity

**DOI:** 10.3390/antiox12111982

**Published:** 2023-11-08

**Authors:** Aimilia Giannaki, Hara Τ. Georgatzakou, Sotirios P. Fortis, Alkmini T. Anastasiadi, Efthimia G. Pavlou, Efrosyni G. Nomikou, Maria P. Drandaki, Angeliki Kotsiafti, Aikaterini Xydaki, Christina Fountzoula, Effie G. Papageorgiou, Vassilis L. Tzounakas, Anastasios G. Kriebardis

**Affiliations:** 1 Laboratory of Reliability and Quality Control in Laboratory Hematology (HemQcR), Department of Biomedical Sciences, School of Health & Caring Sciences, University of West Attica (UniWA), 12243 Egaleo, Greece; agiannaki@uniwa.gr (A.G.); cgeorgatz@uniwa.gr (H.T.G.); sfortis@uniwa.gr (S.P.F.); aanastasiadi@uniwa.gr (A.T.A.); epavlou@uniwa.gr (E.G.P.); efipapag@uniwa.gr (E.G.P.); 2 Department of Biochemistry, School of Medicine, University of Patras, 26504 Patras, Greece; vtzounakas@upatras.gr; 3 Blood Bank and Hemophilia Unit, Hippokration Hospital, 11527 Athens, Greece; aima@hippocratio.gr; 4 Thalassemia and Sickle Cell Unit, Expertise Center of Hemoglobinopathies and Their Complications, Hippokration General Hospital, 11527 Athens, Greece; thalassemia@chaniahospital.gr (M.P.D.); ankotsiafti@uth.gr (A.K.); xydaki@hippocratio.gr (A.X.); 5 Laboratory of Chemistry, Biochemistry and Cosmetic Science (ChemBiochemCosm), Department of Biomedical Sciences, School of Health & Caring Sciences, University of West Attica (UniWA), 12243 Egaleo, Greece; chfountz@uniwa.gr

**Keywords:** sickle cell disease, L-glutamine, RDW, coagulation, inflammation, oxidative stress

## Abstract

Sickle cell disease (SCD) is heterogeneous in terms of manifestation severity, even more so when in compound heterozygosity with beta-thalassemia. The aim of the present study was to stratify β^S^β^+^ patient blood samples in a severity-dependent manner. Blood from thirty-two patients with HbS/β-thalassemia compound heterozygosity was examined for several parameters (e.g., hemostasis, inflammation, redox equilibrium) against healthy controls. Additionally, SCD patients were a posteriori (a) categorized based on the L-glutamine dose and (b) clustered into high-/low-RDW subgroups. The patient cohort was characterized by anemia, inflammation, and elevated coagulation. Higher-dose administration of L-glutamine was associated with decreased markers of inflammation and oxidation (e.g., intracellular reactive oxygen species) and an altered coagulation profile. The higher-RDW group was characterized by increased hemolysis, elevated markers of inflammation and stress erythropoiesis, and oxidative phenomena (e.g., membrane-bound hemoglobin). Moreover, the levels of hemostasis parameters (e.g., D-Dimers) were greater compared to the lower-RDW subgroup. The administration of higher doses of L-glutamine along with hydroxyurea seems to attenuate several features in SCD patients, probably by enhancing antioxidant power. Moreover, anisocytosis may alter erythrocytes’ coagulation processes and hemolytic propensity. This results in the disruption of the redox and pro-/anti-inflammatory equilibria, creating a positive feedback loop by inducing stress erythropoiesis and, thus, the occurrence of a mixed erythrocyte population.

## 1. Introduction

Sickle cell disease (SCD) is one of the most common and severe monogenic disorders worldwide and is estimated to affect ~300,000 infants every year. SCD, which Pauling characterized as a “molecular disease” in 1949, occurs due to a point mutation in the β-globin gene that leads to the production of hemoglobin S (HbS). Due to its altered biophysical properties, the latter can polymerize under hypoxic conditions, and the fibers formed change red blood cell (RBC) deformability features [1]. This is followed by a cascade of events, including vessel occlusion and hemolysis, which are responsible for the symptomatology of the disease, such as pain crises, and even organ damage [2]. Despite its monogenic basis, some clinical phenotypes of SCD present extreme variability, compelling scientists to propose that it concerns a monogenic disease with a polygenic phenotype [3]. The most frequent and most studied form of SCD is the homozygous state for the β^s^ mutation, while other forms include compound heterozygosity for HbS and thalassemias. Nevertheless, even within the distinct genotypes, there is a wide range of manifestation severity.

The variable symptom severity has been linked to several distinct blood parameters. RBCs with low deformability present increased adhesive properties, with both features being indisputable contributors to the disease sequelae [4,5,6]. The levels of microRNAs have been also linked to patient symptomatology [7], while free heme has been similarly discussed as a potential biomarker [8]. Of note, a cluster of seventeen circulating molecules, including hematological parameters (e.g., monocytes, MCV) and serum markers (e.g., lactate dehydrogenase, bilirubin), has been found to correlate with morbidity and mortality in SCD [9]. More recently, the presence of mitochondria-retaining RBCs and reticulocytes, as well as free mitochondrial DNA, has been associated with well-known contributors to the disease manifestations, including hemolysis and inflammatory reactions [10,11,12].

As stated above, the β^S^ mutation can be combined with other mutations in compound heterozygosity. While HbS/β^0^ thalassemia clinically resembles the homozygous sickle cell disease state, HbS/β^+^ thalassemia’s manifestations vary depending on the specific allele [13], and, although it is considered a mild disease, this is not always the case [14]. The present study aimed to find a stratifying parameter to evaluate the variability in disease-manifestation-related parameters in the blood of HbS-β^+^ thalassemia compound heterozygotes, which compose a less studied SCD subgroup.

## 2. Materials and Methods

### 2.1. Subjects

Thirty-two patients with HbS/β-thalassemia compound heterozygosity (HbS-β^+^) and twenty age- and sex-matched healthy controls were included in the study. All HbS-β^+^ patients were receiving folate and most were under treatment with hydroxyurea with/without L-glutamine, while some of them were transfusion-dependent. L-glutamine was consumed by the patients as a food supplement (dose: 16 ± 6 g/day; Glutamine DB EXTRA supplement). The exclusion criteria were as follows: vaso-occlusive crisis 1 month prior to onset of L-glutamine, malignancy, known peptic ulcer, and intolerance to any of the ingredients. Whole blood samples from both patients and controls were collected in ethylenediaminetetraacetic acid (EDTA) and 3.2% sodium citrate blood collection tubes (BD Vacutainer Blood Collection Tubes, BD Biosciences, San Jose, CA, USA).

### 2.2. Material Supplies

All materials and common chemicals were obtained from Sigma-Aldrich (Munich, Germany) unless otherwise stated. Antibodies used for flow cytometry experiments were obtained from BD Biosciences (San Jose, CA, USA). The Zymuphen™ EV activity kit (Hyphen Biomed, Neuville-sur-Oise, France) was used for the measurement of extracellular vesicles’ procoagulant activity. The ECL Western blot detection kit was from GE Healthcare. Antibodies against Hb (CR8000GAP) were from Europa Bioproducts, (Ipswich, UK) while the antibody against 4.1 R was kindly provided by Prof. J. Delaunay (Laboratoire d’ Hématologie, d’Immunologie et de Cytogénétique, Hopital de Bicetre, Le Kremlin-Bicetre, France). HRP-conjugated antibodies to rabbit IgGs were from GE Healthcare (Chicago, IL, USA) and those to goat IgGs were from Sigma-Aldrich (St. Louis, MO, USA).

### 2.3. Hematological and Biochemical Analysis

Classical hematological analysis was performed using a Siemens Advia 2120i Hematology Analyzer. Biochemical analysis of serum components (urea, creatinine, uric acid, glucose, cholesterol, triglycerides, calcium, phosphorus, potassium, sodium, chlorine, magnesium, iron, ferritin, B12, folate, proteins, albumin, serum glutamyl oxalate transaminase, serum glutamyl pyruvate transaminase, gamma-glutamyl transferase, alkaline phosphatase, total, direct and indirect bilirubin, total creatine phosphokinase, amylase, lactate dehydrogenase, and vitamin D) was conducted using the automatic Clinical Chemistry Analyzer ARCHITECT C16000 (Abbott, Chicago, IL, USA). C-reactive protein levels were determined in the Architect C8000 analyzer by a commercial kit (Abbott Laboratories (Hellas), Athens, Greece). HbS and HbF levels were evaluated by the fully automated VARIANT II Hemoglobin Testing System (BioRad, Hercules, CA, USA).

### 2.4. Hemolysis and Redox Parameters

Spontaneous hemolysis (levels of plasma Hb) was calculated by spectrophotometry using Harboe’s method, followed by Allen’s correction and normalization to hematocrit and intracellular Hb levels. The propensity of erythrocytes for osmotic lysis was evaluated upon exposure to ascending NaCl concentrations. The antioxidant capacity of plasma (total, TAC; uric acid-dependent, UAdAC; and uric acid-independent, UAiAC) was measured via the method of Benzie and Strain [15]. Briefly, plasma with and without uricase treatment, mixed with the ferric reducing/antioxidative power (FRAP) solution, was incubated at 37 °C for 4 min and absorbance was measured at 593 nm. Intracellular mean fluorescence and the percentage of reactive oxygen species (ROS)-positive RBCs were detected in a FACSCanto II Cytometer (BD Biosciences, San Jose, CA, USA) by using the fluorescent probe CM-H_2_DCFDA (Invitrogen, Molecular Probes, Eugene, OR, USA) [16].

### 2.5. Hemostasis Parameters

A secondary hemostasis screening test, including the measurement of prothrombin time (PT-INR), activated partial thromboplastin clotting time (APTT), and fibrinogen and D-dimer values, as well as the levels of von Willebrand and factor VIII, was performed as previously described [16]. Quantitative determination of the thrombin/antithrombin III complex (TAT) in human plasma was assessed by enzyme immunoassays (Siemens Healthineers, Erlangen, Germany). Thrombin time (TT) was measured by STA Thrombin reagent (Diagnostica Stago, Parsippany, NJ, USA).

### 2.6. Membrane Isolation and Immunoblotting

RBC membranes were isolated by hypotonic (5 mmol/L sodium phosphate buffer) lysis of RBCs. Membrane aliquots were immune-probed for integral and membrane-bound proteins by using horseradish-peroxidase-conjugated secondary antibodies and enhanced chemiluminescence development. Semi-quantification of protein bands was performed by scanning densitometry (Gel Analyzer v.1.0, Biosure, Athens, Greece).

### 2.7. Statistical Analysis

For statistical analysis, the Statistical Package for Social Sciences (IBM SPSS Software; version 26.0 for Windows IBM Corp., Armonk, NY, USA; administrated by UniWA and the University of Patras) was used. Patients were a posteriori categorized using the information regarding L-glutamine consumption or a two-step cluster analysis (in the case of RDW). The differences between groups were evaluated by parametric and non-parametric tests according to the distribution profile of each parameter. Significance was accepted at *p* < 0.05.

## 3. Results

### 3.1. Variation from Controls

The first step of the study was to ensure that our patient cohort presented the anticipated divergence from the healthy controls. As expected, SCD patients were characterized as anemic due to decreased RBC counts, intracellular Hb, and hematocrit, but an increased red cell distribution width (RDW) (Table 1). Besides the higher levels of HbF and HbS, more reticulocytes were found in the SCD samples. While the complete number of white blood cells did not differ from that in the controls, the proportion of monocytes and basophils was found increased. The biochemical analysis showed that markers of hemolysis, like bilirubin and lactate dehydrogenase, along with markers of inflammation (ferritin, C-reactive protein) and liver function (e.g., alkaline phosphatase and gamma-glutamyl transferase), also presented higher values in the serum of SCD patients compared to controls. Several hemostasis and coagulation parameters, including the levels of D-dimers and von Willebrand factor, and the procoagulant activity of EVs, were elevated in the disease cohort. Lastly, the free Hb and plasma antioxidant capacity were higher in SCD patients, along with the ROS cargo of their RBCs, while osmotic hemolysis was lower (Table 1).

### 3.2. Glutamine-Based Categorization

The patient cohort varied in terms of therapy; therefore, the next step of the present study was to evaluate the effect of the dose of orally consumed L-glutamine to hydroxyurea-treated SCD patient samples. Transfusion-dependent patients were excluded from this categorization so as to consider only the effect of the dose of L-glutamine supplementation. The subgroup that consumed 15 g or more of glutamine per day presented elevated neutrophils and monocytes but decreased lymphocytes (Table 2). Regarding RBC values, mean corpuscular hemoglobin (MCH) and mean corpuscular volume (MCV) were reduced in the high-intake group, along with the presence of nucleated erythrocytes. The levels of inflammation-related molecules, like iron, ferritin, and immunoglobulins, were diminished in the same group, while markers of hemolysis, including lactate dehydrogenase and hemolysis percentage, presented a non-statistically significant trend for lower levels (0.05 < *p* < 0.10). It should be noted that the levels of HbF were decreased after supplementation with >15 g glutamine. Interestingly, the thrombin–antithrombin complex, fibrinogen, and D-dimers were decreased in the high-intake cohort, a result that also arose in oxidative stress parameters, like ROS accumulation and membrane-bound hemoglobin dimers (Table 2).

### 3.3. RDW-Based Categorization

**Figure 1:**
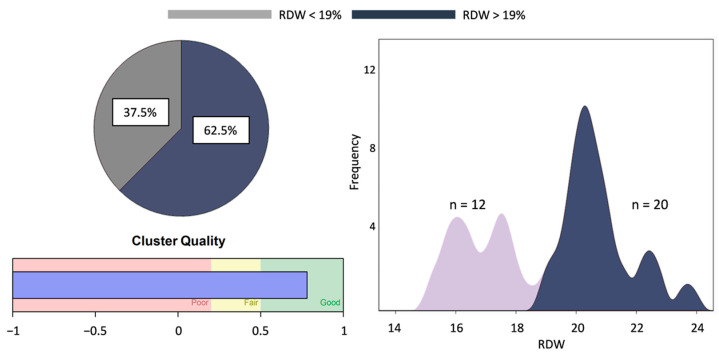
Cluster analysis revealed two subgroups stratified by RDW values.

Through clustering analyses, we sought to categorize all SCD patients, regardless of the choice of therapy, based on one of the parameters measured. The analysis outcome revealed that RDW had good potential in dichotomizing our heterogeneous patient group (Figure 1). A cut-off value of RDW = 19% split our cohort into two distinct subgroups with minimal overlap (Figure 1) and significant differences. To initially examine the effect of L-glutamine consumption upon RDW stratification, we checked the presence of high- and low-dose glutamine recipients in the new cohorts. There was representation of both L-glutamine groups in both RDW categories (six low- and four high-dose in the high-RDW group vs. four low- and five high-dose in the low-RDW group, dose of glutamine: 13.5 ± 6.3 vs. 16.7 ± 7.1 g, respectively, *p* = 0.315).

**Figure 2:**
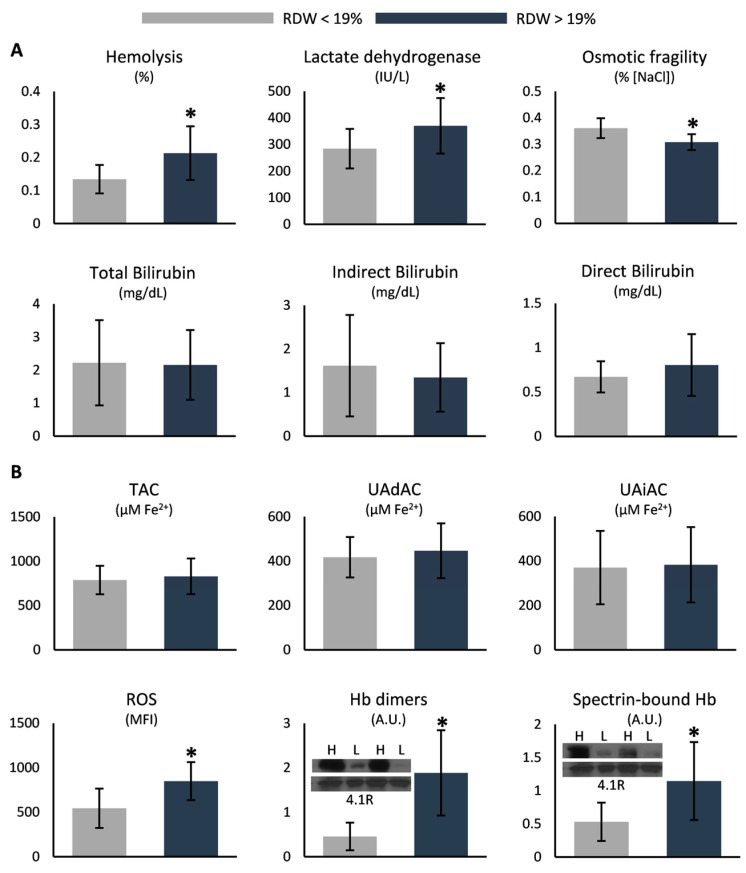
Differences in (**A**) hemolysis markers and (**B**) redox parameters between SCD samples stratified by their RDW values. Inserts: immunoblots of selected samples for high (H) and low (L) RDW subgroups. The 4.1R protein was used as an internal loading control. TAC: total antioxidant capacity; UAdAC: uric-acid-dependent antioxidant capacity; UAiAC: uric-acid-independent antioxidant capacity; ROS: reactive oxygen species; MFI: mean fluorescence intensity; Hb: hemoglobin. (*) *p* < 0.05.

Regarding RBC parameters, the levels of intracellular Hb were lower in the group of RDW > 19% (10.8 ± 1.8 vs. 9.5 ± 1.1 g/dL, lower RDW vs. higher RDW, *p* = 0.017), in contrast to the percentages of reticulocytes and nucleated RBCs, which exhibited the opposite result (reticulocytes: 4.3 ± 1.1 vs. 9.4 ± 4.2%; nucleated RBCs: 5.4 ± 5.1 vs. 24.7 ± 15.2%, lower RDW vs. higher RDW, *p* = 0.01 and 0.005, respectively). While bilirubin did not differ between the two groups, the levels of hemolysis and serum lactate dehydrogenase were higher in the patients with increased RDW (Figure 2A). Osmotic hemolysis presented lower values in the same group (Figure 2A), while extracellular K^+^ was elevated (4.1 ± 0.2 vs. 4.4 ± 0.3 mmol/L, lower RDW vs. higher RDW, *p* = 0.006). Oxidative stress markers, like intracellular ROS accumulation and the binding of Hb to the membrane and the cytoskeleton, were also elevated in patients of higher RDW compared to their counterparts (Figure 2B). Nonetheless, plasma antioxidant capacity was similar between the two groups (Figure 2B).

**Figure 3:**
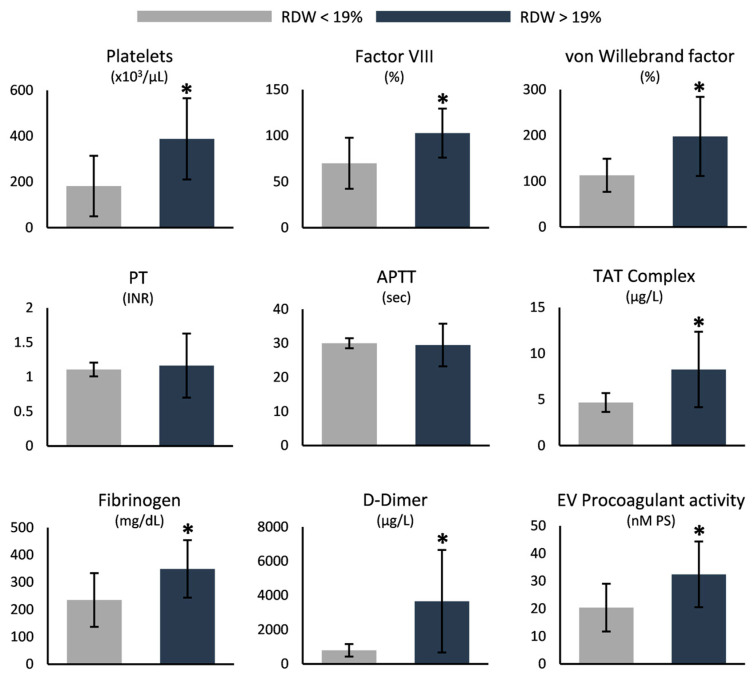
Differences in hemostasis and coagulation parameters between SCD samples stratified by their RDW values. PT: prothrombin time; INR: international normalized ratio; APTT: activated partial thromboplastin time; TAT: thrombin–antithrombin; EV: extracellular vesicles; PS: phosphatidylserine. (*) *p* < 0.05.

Hemostasis and coagulation parameters were notably dissimilar between the two groups, with most of them, including platelets, von Willebrand factor, fibrinogen, and D-dimers, exhibiting increased values in the higher-RDW group (Figure 3). Of note, neither the prothrombin time nor the activated partial thromboplastin time were altered (Figure 3).

**Figure 4:**
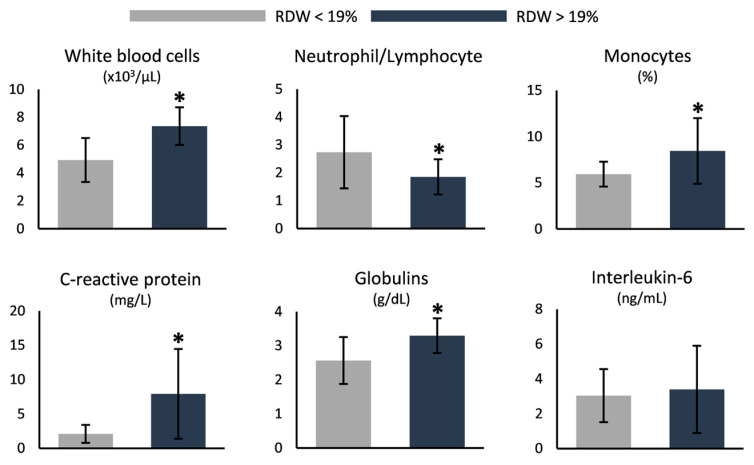
Differences in indicators of inflammation between SCD samples stratified by their RDW values. (*) *p* < 0.05.

The white blood cell count was higher in the group with increased RDW, with monocytes following the same pattern but the neutrophil to lymphocyte ratio being inferior compared to the lower-RDW group (Figure 4). In the same context, markers of inflammation, like C-reactive protein and globulins, were elevated in the group of RDW > 19%, even though interleukin-6 was unaffected (Figure 4). Finally, the levels of serum albumin were lower in the same group (4.7 ± 0.3 vs. 4.3 ± 0.3 g/dL, lower RDW vs. higher RDW, *p* = 0.003).

## 4. Discussion

Compound heterozygotes are a highly heterogeneous group of SCD patients, mainly owing to the nature of the second mutation in β-genes that accompanies β^S^. The combination of the alleles is expected to influence both hallmarks of the disease’s manifestations, as well as the overall clinical picture. In this context, we hereby present (a) data regarding the impact of the dose of glutamine consumption upon markers of oxidation, inflammation, and coagulation and (b) the potential of RDW to categorize heterogeneous patient cohorts with regard to familiar features of SCD-related phenomena, such as free hemoglobin, oxidative stress, hemostasis, and pro-inflammatory molecules. Both analyses of the research were carried out in a compact patient cohort that was distinguished from healthy controls as expected.

### 4.1. Higher Dose of L-Glutamine Is Protective in Terms of Oxidation, Coagulation, and Inflammation

The first drug to be approved for SCD was hydroxyurea, which leads to the elevation of HbF levels, diluting, in this way, HbS and decreasing the probability of its polymerization [17]. In 2017, L-glutamine was also approved as an SCD medication, and it attenuates the disease’s symptoms when administered either alone or in combination with hydroxyurea [18]. Glutamine can strengthen the erythrocytic antioxidant power by contributing to glutathione synthesis, as well as the synthesis of nicotinamide adenine dinucleotide (NAD^+^) and its reduced form, NADH [19]. In this context, the currently presented low oxidative markers in the group that was characterized by higher doses of L-glutamine were rather anticipated.

While the introduction of L-glutamine as an SCD therapeutic agent was based on its potential antioxidant effect, it should be noted that L-glutamine is a quite versatile amino acid. The orally administered L-glutamine supports L-arginine production, the bioavailability of which is reduced in SCD patients but is vital for the formation of nitric oxide (NO) [19]. Among its other roles, NO inhibits hemostatic activation and adhesion molecules [20]. Interestingly, in our study, some parameters of coagulation and fibrinolysis, like the thrombin–antithrombin complex and D-dimers, were reduced after higher L-glutamine supplementation. Accordingly, SCD patients treated with oral L-glutamine present reduced adhesion of sickle erythrocytes to endothelial cells [21]. In a different disease setting, namely hypertension, there are drugs that lead to a D-dimer decrease and, at the same time, NO elevation [22]. Whether the production of NO after glutamine administration is responsible for the current observation is not yet clear.

It should not be omitted that glutamine is an important amino acid for the metabolism of immune cells and a significant modulator of leukocyte function. Among white blood cells, neutrophils –increased in the current study– consume glutamine at the highest rates [23], to support their energy and redox metabolism. Nonetheless, glutamine is also known for its anti-inflammatory roles, since it suppresses the NFκB and STAT pro-inflammatory pathways [24]; therefore, in a disease characterized by a steady inflammatory state, like SCD, its administration could be beneficial in terms of inflammatory phenotypes. Indeed, our results support a slightly differentiated inflammation profile when glutamine is present in higher doses, as evidenced by the lower levels of lymphocytes, and immunoglobulins, which might both be increased in the specific disease setting [25,26], as well as the decreased levels of inflammation markers like ferritin.

While hemolysis parameters did not differ significantly between the two subgroups, there was a trend for improved lysis profiles after high-dose glutamine consumption, a finding consistent with a recent study that examined the effect of prolonged glutamine administration on the clinical, hematological, and biochemical features of SCD patients [27]. Another indicator of disease severity that was significantly reduced was the presence of nucleated RBCs, which reflects increased erythropoietic drive or bone marrow necrosis [28]. On the other hand, the observed lower levels of HbF in the higher-dose group are a finding that needs further investigation, since, to our knowledge, there is not a reported relation between glutamine administration and HbF production. Of note, the secondary outcomes of a recent clinical trial [18] regarding glutamine supplementation/HbF production are still anticipated.

### 4.2. Increased RDW Is Linked to Markers of Disease Severity

RDW is a hematological parameter that seems to characterize systemic abnormalities, since it has been suggested as a potential biomarker in several pathological states, including cardiovascular diseases [29], autoimmunity [30], and cancer [31]. Sickle cell disease is not absent from this list, since, in β^S^β^S^ patients, low RDW has been found to be associated with milder clinical manifestations and improved hemoglobin parameters [32]. It was recently suggested that oxidative stress affects RDW [33]; therefore, redox disequilibrium could lie behind the constant emergence of the biomarker potential of this parameter.

In fact, in the current study, oxidative stress, as evidenced by ROS accumulation and Hb binding to the membrane, was increased in the higher-RDW group. Intracellular ROS have a significant impact on the physiology of RBCs. The membrane and the cytoskeleton are the two main sites of oxidative stress manifestation, through the carbonylation of proteins, peroxidation of lipids, and attachment of hemichromes [34]. All these make RBCs more prone to lysis, in consistency with our current hemolysis findings. Notably, osmotically induced hemolysis was inversely associated with the levels of RDW. It is well known that in hemoglobinopathies, such as thalassemic syndromes and SCD, the affected cells, mostly in the disease state but also in heterozygous conditions, are less fragile following osmotic stress. Moreover, at least in the beta-thalassemia trait, the higher the severity of the mutation, the more osmotically resistant RBCs are [35], while the irreversibly sickled cells have been linked to lower osmotic fragility in SCD due to their excessive damage [36].

The RDW index was recently suggested to be associated with the interactions of RBCs with vessels [37]. The size of erythrocytes, as indicated by RDW, may contribute to the platelet margination effect by altering the dynamic behavior in the bulk flow [37,38]. Platelets can then interact with adhesive proteins, including von Willebrand factor, aggregate, and become activated. The initiation of the coagulation cascade, supported by the elevated levels of factor VIII, thrombin, and fibrinogen, can then lead to the formation of thrombus, which can be then lysed via fibrinolysis, as indicated by the increased levels of D-dimers. In addition, the higher hemolysis in those with increased anisocytosis can lead to the generation of free heme [39], which can act in a prothrombotic way, as recently presented in detail [40]. Lastly, the increased levels of extracellular vesicles that expose phosphatidylserine on their exterior can also activate the coagulation cascade due to the negatively charged surface that enhances the activation of prothrombin to thrombin [41].

In previous studies, RDW has been closely associated with inflammation markers, like interleukins and C-reactive protein [42]. Accordingly, in the current study, the high-RDW group was associated with a slight increase in some inflammation markers, like leukocyte counts, C-reactive protein, and immunoglobulins. Monocytes, which have been reported to be elevated in SCD and were also increased in the currently presented high-RDW subgroup, are often modulated into inflammatory monocytes [43], which intensifies the production of pro-inflammatory molecules in SCD. Inflammation and the altered macrophages can alter both erythropoiesis and erythrocyte survival, which can lead to a mixed RBC population in circulation, mirrored by RDW [44]. Consequently, the modified inflammatory profile could be attributed to the increased extracellular hemoglobin (which acts as a damage-associated molecular pattern [45]), the high oxidative stress [46], or the activation of hemostasis [47] in the high-anisocytosis group, and its occurrence might positively feedback the observed size heterogeneity in the circulating RBCs [48]. To support this, the increased presence of nucleated RBCs and reticulocytes in the high-RDW group is suggestive of stress erythropoiesis [44].

Our study has several limitations. First of all, the a posteriori stratification used both for L-glutamine and RDW renders our findings essentially descriptive. In fact, and since the presented study was not designed as a clinical trial, the examined groups were not constructed beforehand but the patients were only studied for a period during which some of them were already under oral L-glutamine consumption, hydroxyurea treatment, or transfusion therapy. In this context, the fact that there are no data regarding the period before the start of L-glutamine consumption limits the potential to better (a) appreciate the effect of glutamine administration and (b) evaluate dose-dependent outcomes. Such information would be of great value for future studies, and especially randomized clinical trials, since it would support the evaluation of the patients’ cohorts both before and after supplement administration, instead of reporting a snapshot. However, we strongly believe that, in light of the few studies that focus on L-glutamine in the context of SCD, and especially the less studied group of β^S^β^+^ compound heterozygotes, the reported data remain valuable and challenging and can give insight into the biochemical and physiological profiles of these patients in relation to food supplement consumption.

## 5. Conclusions

The present study suggests that glutamine in combination with hydroxyurea has a beneficial effect on β^S^β^+^ blood parameters, probably due to its implication in enhancing the antioxidant defenses. Moreover, despite the high variability in several parameters, RDW seems to adequately subcategorize patients, with its levels being related to well-known contributors to the disease, such as hemolysis, oxidative stress, inflammation, and coagulation. While it is true that “RDW cannot be regarded as the ‘panacea’ of the new century”, as perfectly stated by Lippi and Plebani [49], it seems to be an informative parameter in heterogeneous conditions such as sickle cell disease.

## Figures and Tables

**Table 1:** Variations in SCD patients from healthy controls.

	Patients	Controls	Normal Range
Age (years)	49.8 ± 11.9	44.7 ± 7.6	
General Blood Test			
White blood cells (×10^3^/μL)	7.6 ± 1.7	6.4 ± 1.3	5.2–12.4
Neutrophils (%)	57.4 ± 9.0	56.8 ± 7.1	40.0–74.0
Lymphocytes (%)	29.3 ± 7.3	31.3 ± 6.5	19.0–48.0
Monocytes (%)	7.5 ± 3.1 *	5.8 ± 1.3	3.4–9.0
Eosinophils (%)	2.5 ± 1.3 *	3.6 ± 1.8	0.0–7.0
Basophils (%)	1.0 ± 0.5 *	0.7 ± 0.3	0.0–1.5
Neutrophil/lymphocyte ratio	2.2 ± 1.0	1.9 ± 0.5	-
Red blood cells (×10^6^/µL)	3.8 ± 0.9 *	5.0 ± 0.4	4.2–6.1
Hemoglobin (g/dL)	10.0 ± 1.5 *	14.2 ± 1.1	12.0–18.0
Hematocrit (%)	31.8 ± 4.4 *	44.0 ± 3.6	37.0–52.0
MCV(fL)	86.4 ± 12.0	88.9 ± 4.0	80.0–99.0
MCH (pg)	27.1 ± 4.2	28.6 ± 1.3	27.0–31.0
MCHC (gr/dL)	31.3 ± 1.3 *	32.2 ± 0.7	33.0–37.0
RDW (%)	19.3 ± 2.3 *	13.1 ± 0.9	11.5–14.5
Platelets (×10^3^/µL)	310.7 ± 189.2	273.4 ± 40.0	130.0–400.0
Mean platelet volume (MPV; fL)	10.0 ± 1.2	9.5 ± 1.4	7.2–11.1
Reticulocyte count (%)	8.0 ± 4.8 *	1.5 ± 0.3	0.5–2.0
HbS (%)	65.9 ± 14.3 *	0.0 ± 0.0	0
HbF (%)	14.3 ± 9.6 *	0.3 ± 0.1	0.8–2
Serum Biochemical Analysis			
Glucose (mg/dL)	89.3 ± 10.1 *	81.5 ± 10.8	70–105
Urea (mg/dL)	28.8 ± 13.7	27.2 ± 5.1	18.0–55.0
Creatinine (mg/dL)	0.78 ± 0.18	0.83 ± 0.09	0.72–1.25
Uric acid (mg/dL)	5.1 ± 1.0	4.7 ± 0.8	3.5–7.2
Cholesterol (mg/dL)	149.4 ± 25.4 *	183.1 ± 22.6	0.0–200.0
Triglycerides (mg/dL)	124.7 ± 53.1	99.7 ± 37.0	0.0–150.0
Calcium (mg/dL)	9.2 ± 0.6	9.3 ± 0.4	8.4–10.2
Phosphorus (mg/dL)	3.4 ± 0.5	3.4 ± 0.5	2.4–4.7
Potassium (mmol/L)	4.3 ± 0.3	4.2 ± 0.2	3.5–5.1
Sodium (mmol/L)	138.6 ± 1.8	139.4 ± 1.8	136.0–145.0
Chlorine(mmol/L)	105.7 ± 1.9	105.8 ± 1.8	98.0–107.0
Magnesium (mg/dL)	2.0 ± 0.3	2.0 ± 0.1	1.60–2.60
Iron (mg/dL)	130.0 ± 96.1	109.4 ± 51.0	50–150 (F); 60–160 (M)
Ferritin (ng/mL)	359.9 ± 215.5 *	61.0 ± 42.3	14.0–233.0 (F); 16.4–293.3 (M)
B12 (pg/mL)	336.3 ± 146.2	371.4 ± 185.0	179.0–1162.0
Folate (ng/mL)	24.1 ± 15.0 *	6.4 ± 2.5	2.5–17.0
Proteins (mg/dL)	7.4 ± 0.6	7.3 ± 0.4	6.40–8.30
Albumin (gr/dL)	4.4 ± 0.3	4.4 ± 0.3	3.50–5.00
SGOT (U/L)	32.4 ± 11.5 *	19.0 ± 6.3	5.0–34.0
SGPT (U/L)	28.8 ± 25.0	22.3 ± 12.6	0.0–55.0
Gamma-glutamyl transferase (U/L)	34.4 ± 24.1 *	19.2 ± 9.6	12.0–64.0
Alkaline phosphatase (ALP; U/L)	78.5 ± 23.1 *	63.1 ± 11.9	40.0–150.0
HDL (mg/dL)	39.9 ± 8.7 *	55.4 ± 14.1	>50
LDL (mg/dL)	84.7 ± 20.9 *	107.8 ± 18.9	<110
Immunoglobulins (g/dL)	3.0 ± 0.7	2.9 ± 0.7	
Total bilirubin (mg/dL)	2.2 ± 1.1 *	0.6 ± 0.2	0.2–1.2
Indirect bilirubin (mg/dL)	1.4 ± 0.9 *	0.3 ± 0.1	0.01–0.9
Direct bilirubin (mg/dL)	0.7 ± 0.3 *	0.2 ± 0.1	0.00–0.30
Lactate dehydrogenase (IU/L)	337.9 ± 102.1 *	184.5 ± 31.7	125.0–220.0
Creatine phosphokinase total (IU/L)	41.8 ± 29.7 *	107.7 ± 80.9	30.0–200.0
Vitamin D (ng/mL)	24.3 ± 11.1	21.4 ± 8.2	30.0–100.0
C-reactive protein (mg/L)	6.0 ± 5.9 *	1.7 ± 1.6	0.0–5.0
Hemostasis–Coagulation Parameters			
Prothrombin time INR	1.1 ± 0.4 *	1.0 ± 0.1	0.8–1.1
APTT (s)	29.7 ± 5.0	29.2 ± 2.9	<36
Fibrinogen (mg/dL)	306.2 ± 115.5	327.9 ± 74.1	180–350
D-Dimer (µg/L)	2552.6 ± 2186.0 *	261.9 ± 98.4	<500
Factor VIII (%)	90.6 ± 31.0 *	123.3 ±17.6	60–140
von Willebrand factor (%)	166.3 ± 82.4 *	110 ± 20.8	60–140
TAT complex (μg/L)	7.0 ± 3.7 *	3.2 ± 0.6	2.0–4.2
EV procoagulant activity (nM PS)	28.0 ± 12.2 *	20.3 ± 8.5	
Hemolysis and Redox Status			
Hemolysis (%)	0.20 ± 0.09 *	0.09 ± 0.08	
Osmotic fragility (% [NaCl])	0.33 ± 0.04 *	0.46 ± 0.02	
Intracellular ROS (MFI)	744.1 ± 258.5 *	480.6 ± 211.2	
Plasma TAC (μM Fe^2+^)	782.6 ± 184.9 *	506.3 ± 109.4	
Plasma UAdAC (μM Fe^2+^)	438.2 ± 106.9 *	306.2 ± 125.1	
Plasma UAiAC (μM Fe^2+^)	344.4 ± 166.2 *	200.0 ± 59.8	

Values are presented as mean ± SD. Bold, (*): *p* < 0.05 patients vs. controls. APTT: activated partial thromboplastin time; A.U.: arbitrary units; EV: extracellular vesicles; HDL: high-density lipoproteins; LDL: low-density lipoproteins; MCH: mean corpuscular hemoglobin; MCHC: mean corpuscular hemoglobin concentration; MCV: mean corpuscular volume; MFI: mean fluorescence intensity; PS: phosphatidylserine; RDW: red cell distribution width; ROS: reactive oxygen species; SGOT: serum glutamic-oxaloacetic transaminase; SGPT: serum glutamate-pyruvate transaminase; TAC: total antioxidant capacity; TAT complex: thrombin–antithrombin complex; UAdAC: uric-acid-dependent antioxidant capacity; UAiAC: uric-acid-independent antioxidant capacity.

**Table 2:** Variations in SCD hydroxyurea-administered patients upon additional supplementation with glutamine of less or more than 15 g/day.

	<15 g/day (*n* = 10)	≥15 g/day (*n* = 9)
Age (years)	50.5 ± 14.7	40.3 ± 14.7
General Blood Test		
White blood cells (×10^3^/μL)	5.7 ± 1.7	6.1 ± 2.0
Neutrophils (%)	55.4 ± 7.0 *	65.8 ± 7.3
Lymphocytes (%)	33.9 ± 6.3 *	22.8 ± 5.8 (33%)
Monocytes (%)	5.2 ± 1.1 *	6.7 ± 1.1
Eosinophils (%)	2.5 ± 1.0	2.4 ± 1.4
Basophils (%)	0.7 ± 0.2	0.8 ± 0.3
Neutrophil/lymphocyte ratio	1.7 ± 0.7 *	3.1 ± 1.2
Red blood cells (×10^6^/µL)	3.4 ± 0.3 (100%)	4.0 ± 1.2 (55%)
Hemoglobin (g/dL)	10.2 ± 1.1 (90%)	10.2 ± 2.2 (66%)
Hematocrit (%)	32.5 ± 2.4 (100%)	32.4 ± 6.6 (66%)
Mean corpuscular volume (MCV; fL)	97.0 ± 11.3 * (30%)	82.8 ± 9.8 (66%)
Mean corpuscular hemoglobin (MCH; pg)	30.5 ± 4.5 * (40%)	26.0 ± 2.8 (66%)
MCH concentration (MCHC; gr/dL)	31.4 ± 1.2 (80%)	31.5 ± 1.6 (77%)
Red cell distribution width (RDW; %)	19.4 ± 2.4 (100%)	18.6 ± 2.7 (100%)
Platelets (×10^3^/µL)	351.7 ± 263.3 (20%)	306.9 ± 201.9 (77%)
Mean platelet volume (MPV; fL)	10.0 ± 1.2 (10%)	9.6 ± 1.2
Reticulocyte count (%)	7.1 ± 1.6 (100%)	8.4 ± 6.7 (100%)
Nucleated red blood cells (%)	19.8 ± 9.8 *	9.8 ± 7.3
HbS (%)	68.9 ± 6.4 (100%)	73.5 ± 9.3 (100%)
HbF (%)	23.2 ± 7.9 * (100%)	10.3 ± 9.8 (100%)
Serum Biochemical Analysis		
Glucose (mg/dL)	88.7 ± 9.6	84.4 ± 6.8
Urea (mg/dL)	27.5 ± 17.2 (20%)	21.6 ± 7.7 (33%)
Creatinine (mg/dL)	0.78 ± 0.12 (20%)	0.73 ± 0.13 (22%)
Uric acid (mg/dL)	5.2 ± 0.7	5.3 ± 1.2 (11%)
Cholesterol (mg/dL)	156.9 ± 24.4	153.3 ± 27.9
Triglycerides (mg/dL)	136.0 ± 30.2 * (20%)	91.2 ± 47.1 (11%)
Calcium (mg/dL)	9.6 ± 0.8 * (20%)	8.9 ± 0.5 (11%)
Phosphorus (mg/dL)	3.5 ± 0.3	3.4 ± 0.4
Potassium (mmol/L)	4.4 ± 0.3	4.2 ± 0.3
Sodium (mmol/L)	139.1 ± 1.4	140.0 ± 1.2
Chlorine (mmol/L)	105.8 ± 1.5	106.3 ± 1.4
Magnesium (mg/dL)	2.0 ± 0.6 (10%)	2.0 ± 0.1
Iron (mg/dL)	106.5 ± 29.1 *	69.4 ± 26.5 (22%)
Ferritin (ng/mL)	338.8 ± 152.5 * (70%)	56.5 ± 36.0 (11%)
B12 (pg/mL)	269.1 ± 85.0 (20%)	311.2 ± 98.7
Folate (ng/mL)	20.5 ± 16.6 (50%)	25.1 ± 14.5 (55%)
Proteins (mg/dL)	7.8 ± 0.3 *	7.0 ± 0.4
Albumin (g/dL)	4.7 ± 0.2	4.5 ± 0.4
Serum glutamic-oxaloacetic transaminase (SGOT; U/L)	27.8 ± 6.8 (10%)	26.4 ± 14.2 (11%)
Serum glutamate-pyruvate transaminase (SGPT; U/L)	19.8 ± 7.3	22.4 ± 15.2
Gamma-glutamyl transferase (U/L)	20.1 ± 14.1	34.5 ± 15.6
Alkaline phosphatase (ALP; U/L)	76.1 ± 14.6	86.7 ± 24.8
High-density lipoproteins (HDL; mg/dL)	41.5 ± 7.8 (80%)	39.7 ± 9.0 (66%)
Low-density lipoproteins (LDL; mg/dL)	88.5 ± 20.1 (10%)	95.6 ± 22.3 (33%)
Immunoglobulins (g/dL)	3.2 ± 0.4 *	2.5 ± 0.6
Total bilirubin (mg/dL)	1.9 ± 0.9 (80%)	1.7 ± 0.8 (66%)
Indirect bilirubin (mg/dL)	1.3 ± 0.8 (50%)	1.1 ± 0.7 (44%)
Direct bilirubin (mg/dL)	0.65 ± 0.13 (100%)	0.58 ± 0.16 (100%)
Lactate dehydrogenase (IU/L)	284.5 ± 57.9 (80%)	251.8 ± 27.8 (77%)
Creatine phosphokinase total (IU/L)	33.4 ± 19.8 (50%)	47.0 ± 35.6 (11%)
Vitamin D (ng/mL)	21.7 ± 6.8 (70%)	22.9 ± 7.8 (66%)
C-reactive protein (mg/L)	4.3 ± 2.4 (10%)	6.7 ± 6.4 (33%)
Hemostasis–Coagulation Parameters		
Prothrombin time INR	1.02 ± 0.06 (10%)	1.08 ± 0.12 (22%)
Activated partial thromboplastin time (APTT; s)	28.2 ± 2.0	29.1 ± 2.9
Fibrinogen (mg/dL)	364.0 ± 79.5 * (40%)	257.1 ± 104.6 (55%)
D-dimer (µg/L)	1187.6 ± 360.9 * (100%)	693.1 ± 307.1 (55%)
Factor VIII (%)	78.2 ± 23.4 (10%)	75.3 ± 33.4 (33%)
von Willebrand factor (%)	126.8 ± 21.2 (20%)	141.1 ± 84.2 (33%)
Thrombin–antithrombin complex (μg/L)	5.8 ± 1.4 * (50%)	4.3 ± 1.3 (22%)
EV procoagulant activity (nM PS)	22.5 ± 7.6	27.2 ± 6.3
Hemolysis and Redox Status		
Hemolysis (%)	0.21 ± 0.08	0.15 ± 0.05
Osmotic fragility (% [NaCl])	0.33 ± 0.06	0.34 ± 0.02
Intracellular reactive oxygen species (MFI)	880.5 ± 194.9 *	585.4 ± 231.4
Plasma TAC (μM Fe^2+^)	778.7 ± 177.5	745.2 ± 211.2
Plasma UadAC (μM Fe^2+^)	417.1 ± 99.7	435.0 ± 95.0
Plasma UaiAC (μM Fe^2+^)	361.5 ± 158.2	310.2 ± 225.9
Membrane-bound hemoglobin dimers (A.U.)	1.51 ± 0.89 *	0.64 ± 0.46

Values are presented as mean ± SD. Bold, (*): *p* < 0.05 glutamine administration <15 g/day vs. glutamine administration ≥15 g/day. The percentages in parentheses indicate the proportion of patient samples with values outside the normal range. A.U.: arbitrary units; EV: extracellular vesicles; MFI: mean fluorescence intensity; PS: phosphatidylserine; TAC: total antioxidant capacity; UAdAC: uric-acid-dependent antioxidant capacity; UAiAC: uric-acid-independent antioxidant capacity.

## Data Availability

All data presented in this study are available upon request.
